# Measurement of an Analyte Concentration in Test Solution by Using Helmholtz Resonator for Biosensor Applications

**DOI:** 10.3390/s19051127

**Published:** 2019-03-05

**Authors:** Yugang Chen, Yong-Hwa Park

**Affiliations:** Department of Mechanical Engineering, Korea Advanced Institute of Science and Technology, Daejeon 34141, Korea; chenyg@kaist.ac.kr

**Keywords:** analyte concentration, indirect measurement, Helmholtz resonator, resonant frequency, glucose sensor

## Abstract

In this paper, an indirect method of measuring an analyte concentration in a test solution using the resonant frequency change of a Helmholtz resonator is proposed, using a novel architecture of Helmholtz resonator filled with two kinds of fluids (fixed fluid and test solution). Since the analyte concentration yields changes of density and sound speed of the test solution, the resonant frequency of the proposed Helmholtz resonator is affected by the analyte concentration of the test solution. From this effect, the analyte concentration of the test solution can be measured by the spectrum of acoustic resonance of the Helmholtz resonator. The experiment was done using a 3D-printed Helmholtz resonator system with an acoustic power source and detectors, which is consistent with analytical results and showed that the analyte concentration can be measured with higher sensitivity compared to conventional cantilever-type sensors. As an example application, the possibility of measuring glucose concentration of human blood was demonstrated, showing higher sensitivity and relatively low frequency range compared to previous resonance based methods.

## 1. Introduction

Over the past several decades, various types of devices and measurement architectures were introduced in the field of biosensors. Sensing methodology based on vibration principles has been studied extensively, since mechanical resonance can be measured easily with relatively low measurement errors. Among the resonance-based sensors, dynamic-mode cantilever sensors have significant advantages over other sensors, in terms of fast response time, high sensitivity, high resolution and low cost of fabrication [1]. They have had broad applications in the fields of chemistry, biotechnology, etc. [2,3,4,5,6]. These sensing systems can be further specialized to act as functional biosensors, e.g., monitoring glucose concentration, whereby the resonant frequency of the cantilever shifts due to the mass loading effect when biomolecules absorb onto the surface of the cantilever treated with specified enzymes [7,8]. Since the methodology mostly relies only on added mass effect on the resonant frequency, improvements of the sensitivity of the cantilever-type sensors are usually realized by shrinking its physical dimension or measuring higher order modes [9,10], which can result in limitations of physical dimension and frequency measurement.

To overcome these limitations, this paper suggests a new type of biosensor utilizing Helmholtz resonance. Helmholtz resonance is the phenomenon of acoustic resonance in a cavity, presented by Hermann von Helmholtz [11]. During the past decades there have been many researches dedicated to designing new-types of Helmholtz resonators in order to extend the resonance frequency bandwidth as well as to improve the performance of transmission losses in the field of noise control [12,13,14,15]. On the other hand, some researchers used Helmholtz resonators (HRs) as sensors for volume estimation of liquids and solids, revealing high accuracy and sensitivity [16,17]. In addition to the design, fluid inside an HR also has a significant effect on the resonant frequency of the HR. Inspired by the resonance principle of HRs, we suggest using an HR to measure the analyte concentration in a test solution contained in the cavity of an HR as a new type of biosensor.

In this paper, an HR filled with fixed fluid (e.g., pure water or air) and test solution (e.g., solution with varying analyte concentration) is presented, whose change of resonant frequency indicates the change of analyte concentration in the test solution. Theoretical derivation of the measurement principle and experimental investigation were conducted, using the measurement of glucose concentration in the test solution as an example. Results showed higher sensitivity and relatively low frequency range compared to previous resonance based methods. Measurement of a practical level of glucose concentration in human blood using the suggested HR is discussed, revealing the possibility of using it as a biosensor.

## 2. Measurement Principle and Architecture

### 2.1. Underlying Theory

**Figure 1:**
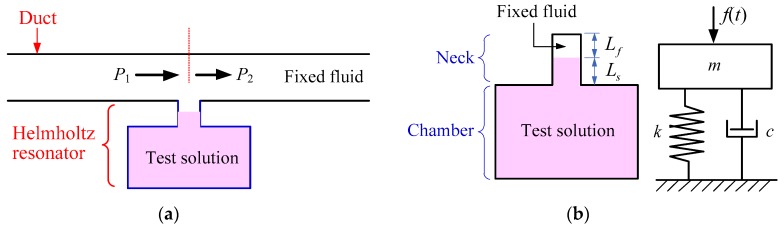
(**a**) The Helmholtz resonator linked with a duct; (**b**) Structure of Helmholtz resonator and its equivalent mass-spring model.

In this paper, for the purpose of measuring the analyte concentration in the test solution, the duct attached with a Helmholtz resonator as a side-branch was proposed as shown in Figure 1a. The test solution fills in the chamber and a portion of the neck of the HR, while the other (upper) part of neck and duct is filled with another fixed fluid. 

Dynamic behavior of the HR can be effectively simplified using a mass-spring system analogy, as shown in Figure 1b. The mass of fluid in the neck (including fixed fluid and test solution) of the HR is equivalent to the mass (*m*), and the adiabatically compressed volume of the fluid in the chamber is equivalent to the spring (*k*). The incident pressure to the HR is equivalent to the input force to the simplified mass-spring system.

The fluids trapped in the neck have a total mass (*m*) of

(1) $$m=\rho_{f}S_{n}L_{f}^{\prime }+\rho_{s}S_{n}L_{s}^{\prime }$$

where $\rho_{f}$ and $\rho_{s}$ are densities of the fixed fluid and the test solution, respectively, and $S_{n}$ is the cross-sectional area of the neck. $L_{f}^{\prime }$ and $L_{s}^{\prime }$ are the equivalent lengths of the fixed fluid and test solution in the neck; they can be computed as $L_{f}^{\prime }=L_{f}+0.36\sqrt{S_{n}}$ and $L_{s}^{\prime }=L_{s}+0.48\sqrt{S_{n}}$ [12].

The stiffness (*k*) resulting from the volume compression of the test solution inside the chamber can be obtained as

(2) $$k=\rho_{s}c_{s}^{2}\frac{S_{n}^{2}}{V}$$

where $c_{s}$ is sound speed of the test solution, and V is the volume of chamber.

Then the resonant frequency of the proposed HR can be calculated by using

(3) $$f_{hr}=\frac{1}{2\pi }\sqrt{\frac{k}{m}}=\frac{c_{s}}{2\pi }\sqrt{\frac{\rho_{s}S_{n}}{\left(\rho_{f}L_{f}^{\prime }+\rho_{s}L_{s}^{\prime }\right)V}}$$

When the HR is attached to a duct as shown in Figure 1a, it functions as a muffler because the acoustic energy is attenuated by mass (*m*) movement in the neck of the HR, which is regulated by the equivalent stiffness (*k*). Thus, resonant frequency of the HR can be acquired by the spectrum of transmission loss (*TL*), and *TL* is defined by the difference between incident acoustic pressure and transmitted acoustic pressure:

(4) $$TL=SPL_{1}-SPL_{2}=20\log_{10}\left(P_{1}/P_{2}\right)$$

where *SPL*_1_ and *SPL*_2_ are acoustic pressure levels in dB scale, and *P*_1_ and *P*_2_ are incident and transmitted acoustic pressures, respectively.

The transmission loss can be analytically obtained by using

(5) $$TL=20\log_{10}\left(\left|1+\frac{\rho_{f}c_{f}}{2S_{d}}\frac{1}{Z_{H}}\right|\right)$$

where $Z_{H}$ is the acoustic impedance of the proposed HR. Detailed derivation and explanation of the transmission loss can be seen in the Supplementary Material.

Equation (3) reveals that resonant frequency of the HR relates directly with the density ($\rho_{s}$) and sound speed ($c_{s}$) of the test solution, which are affected by the analyte concentration in the test solution. Here we take the glucose (sugar) solution as an example. The density of water and pure glucose at 20 °C are 998 kg/m^3^ and 1540 kg/m^3^, respectively. Therefore, the density of glucose solution increases as the glucose concentration increases. In addition to the increase of the density, sound speed in the aqueous solution increases as glucose concentration increases [18]. Since the compressibility of individual glucose molecule is much smaller than that of water, the adiabatic compressibility of the glucose solution ($\beta_{s}$) can be expressed as

(6) $$\beta_{s}=\beta_{0}(1-\Phi )$$

where $\beta_{0}$ is the adiabatic compressibility of water and its value at 20 °C is given by $\beta_{0}=4.565\times 10^{-10}{\mathrm{Pa}}^{-1}$, Φ is the fractional volume of glucose.

Then sound speed ($c_{s}$) in the glucose solution can be obtained by the expression [19]:

(7) $$c_{s}=\frac{1}{\sqrt{\beta_{s}\rho_{s}}}$$

Density and sound speed of water, and 34.9 wt % glucose solution at 20 °C were listed in [19], as shown in Table S1 in the Supplementary Material. By using Equation (7) together with the reference data, density and sound speed of the glucose solution with a concentration of 4.9 wt %, 9.7 wt %, 15.9 wt %, and 30.4 wt % can be reasonably obtained, as listed in Table S1, which shows the increase of both density and sound speed as the glucose concentration increases.

### 2.2. Measurement Principle and Device Architecture

**Figure 2:**
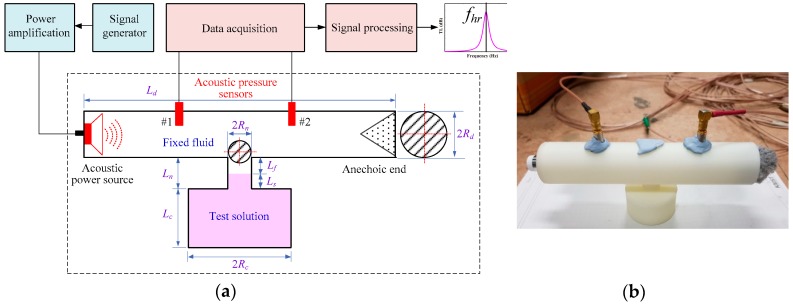
(**a**) Schematic illustration of analyte concentration measurement in the test solution; (**b**) Device architecture of the duct linked with a Helmholtz resonator.

From the underlying theory mentioned above, the resonant frequency of the proposed HR was related with the analyte concentration of the test solution. Considering using an HR to measure the analyte concentration in the test solution, the measurement architecture is shown in Figure 2a, consisting of an HR and duct linked to each other. Two acoustic pressure sensors were used to measure the incident and transmit acoustic pressures in the duct, excited by the acoustic power source at the incident end of the duct. The outlet end of the duct was defined as an anechoic end having full acoustic absorption.

A duct integrated with a side-branch HR was manufactured by 3D printing (Figure 2b), using the geometry and dimensions shown in Table S2 in the Supplementary Material. A small wireless speaker was placed at the incident end of the duct as an acoustic power source, and pressure sensors were used to measure the acoustic pressures of point 1 and point 2, with a frequency range of 10 Hz–20 kHz and a dynamic range of acoustic pressure level of 28–140 dB. Acoustic absorption material (polyester fiber) was used to cover the outlet end of the duct as the anechoic boundary.

In order to validate the underlying theory and experiment setup, a homogenous HR with only air inside was tested. Sine sweep technique was used to generate acoustic pressure within a frequency range of 500–1000 Hz, then the steady-state incident and transmitted acoustic pressures (*P*_1_ and *P*_2_) were measured by pressure sensors number 1 and number 2, respectively. Then the spectrum of transmission loss was calculated by Equation (4) from the measurement of acoustic pressures. Experimental and analytical results are shown in Figure S1 in the Supplementary Material. The experimental result of the resonant frequency was 747 Hz and the analytical result was 735.9 Hz. Relative error between experimental and analytical result was about 1.49%, showing quite a good agreement. The errors come from the fabrication errors of 3D-printed structure and the possible influence of the environment temperature.

## 3. Results

### 3.1. Measurement of Glucose Concentration in Test Solutions with Various Concentrations

Test solutions with different glucose concentrations of 4.9 wt %, 9.7 wt %, 15.9 wt % and 30.4 wt % were made by using an electronic scale to add a controlled amount of glucose into the water in the beaker, then were injected into the cavity of the HR by the injector, as shown in Figure S2 in the Supplementary Material. The fixed fluid selected was air.

**Figure 3:**
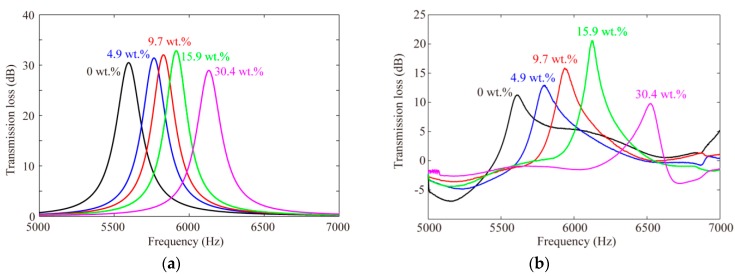
Spectrum of transmission loss under different glucose concentrations. (**a**) Analytical results; (**b**) experimental results.

By using the values of density and sound speed in Table S1 and the dimensions in Table S2, the spectrum of transmission losses under the condition of different glucose concentrations were obtained as shown in Figure 3a. Corresponding values of resonant frequency are listed in Table 1.

Referring to the analytical results, the frequency range of the acoustic power source was selected to be 5000–7000 Hz, in order to observe the resonant frequency of the HR sufficiently. By using a sine sweep technique, acoustic pressures were measured by two acoustic pressure sensors. Then transmission loss spectrum of the HR filled with air as fixed fluid and test solutions of different glucose concentrations were obtained using Equation (4), as shown in Figure 3b. Distinct peaks appeared in the spectrum of transmission loss, and an obvious frequency shift to a higher frequency was observed.

Resonant frequencies obtained by analytical approach and experiment are listed in Table 1 for comparison. In the concentration range of 0–30.4 wt %, the relative errors between analytical results and experiment results were 0.21–5.98%; the relative error tended to be higher for the higher glucose concentration. The calculation of density and sound speed for higher concentration may need to be improved in a higher frequency range. To improve the analytical model to match the measured results, the precise values of density and sound speed need to be measured directly for different glucose concentrations. On the other hand, in spite of the influence of temperature change and evaporation of water on density and sound speed of the glucose solution during the experiment process, the results of the two approaches agreed quite well. Most important of all, obvious frequency increases were observed in both the analytical and experimental results.

### 3.2. Measurement of Practical Glucose Concentration of Human Blood Glucose Level in an Aqueous Solution

For human blood glucose level detection, although there are many substances in human blood and their concentrations may also have an effect on the resonant frequency of the proposed HR, the change of glucose concentration occurs much faster than that of other substances in a daily life time frame. Specifically, the glucose level in human blood changes about 70–160 mg/dL during every meal, whereas other substances such as fat, cholesterol, etc. change very slowly in that time frame. Glucose concentration can be considered as a dominant variable, and human blood can be simplified to be the aqueous solution with a varying glucose concentration over a short term (for example, a week), meaning at least one sensor calibration a week.

For people with diabetes, blood glucose level is recommended to be controlled between 72 and 126 mg/dL before meals, and under 162 mg/dL for people with type 1 diabetes or 153 mg/dL for people with type 2 diabetes after having meals [20]. By the assumption mentioned above that human blood can be simplified to a glucose solution, the corresponding density and sound speed of a glucose solution in the cases of several practical glucose concentrations were obtained, as shown in Table 2.

**Figure 4:**
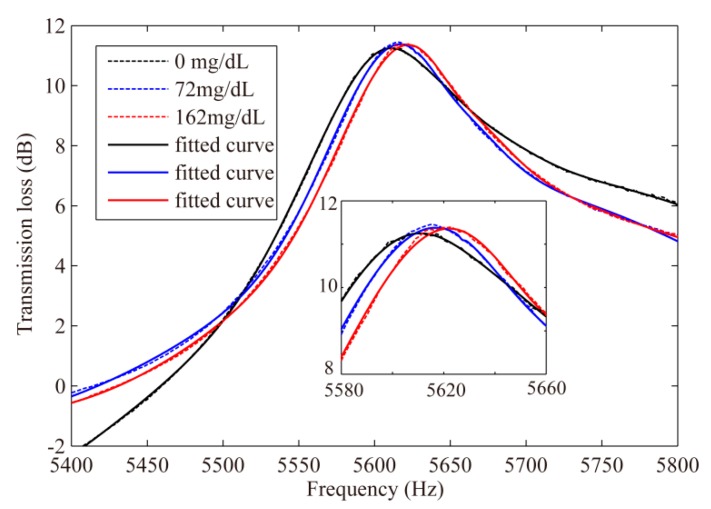
Spectrum of transmission loss at human blood glucose levels obtained by experiment.

Using the parameters in Table 2, resonant frequencies were obtained by the analytical approach with Equation (3) as 5596, 5601 and 5607 Hz with glucose concentrations of 0, 72 and 162 mg/dL, respectively, as listed in Table 2. Figure 4 shows the experimental results (original results and curves after fitting) by using the experiment setup described in the previous sections, with the values listed in Table 2. Noticeable frequency increases were observed as the glucose level increased.

## 4. Discussion

Referring to the sensitivity of liquid density sensor using micro-cantilevers [21], the absolute sensitivity (unit: Hz/(kg/m^3^)) can be defined as the ratio of resonance frequency shift to the solution density variation:

(8) $$S_{a}=\frac{\partial f}{\partial \rho_{s}}$$

and the relative sensitivity (dimensionless index) can be defined as:

(9) $$S_{r}=\frac{\partial f}{\partial \rho_{s}}\frac{\rho_{s0}}{f_{0}}$$

where *f* is the resonant frequency, $\rho_{s}$ is the density of the test solution, and *f*_0_ and $\rho_{s0}$ are the reference resonant frequency and solution density, respectively.

In this example, absolute sensitivity is $S_{a}=11.99\;{\mathrm{Hz}/(\mathrm{kg}/m}^{3})$ and relative sensitivity is $S_{r}=2.14$ near the resonant frequency 5600 Hz. The absolute sensitivity in this example is larger than that of the rectangular micro-cantilever beam with length of 1.5 mm in [21], whose absolute sensitivity is lower than 6.966 Hz/(kg/m^3^) at the frequency range near 10,000 Hz. What’s more, the relative sensitivity is much higher than the traditional cantilever biosensors based on added mass effect of liquid on the resonant frequency of the vibrating beam (lower than 0.5 in most cases) [22].

For the measurement of the practical glucose concentration of human blood glucose level, in this case the absolute sensitivity was obtained as 0.067 Hz/(mg/dL). Considering the low operating frequency range within 6 kHz, it is quite sensitive to distinguish the practical glucose concentration at human blood glucose level. Moreover, when we use the high precision acoustic power source and high precision frequency measurement (e.g., frequency resolution of 0.1 Hz using microelectronic digital signal processing (DSP) devices), the proposed sensing architecture in this paper is able to quantify *R* = 0.1/0.067 = 1.49 mg/dL as the smallest measurement precision of glucose concentration. In previous methods, by using a microwave-based sensor and a micro-strip ring resonator, respectively, the corresponding values were 50 mg/dL [23] and 40 mg/dL [24], respectively.

In the current design of this paper, the amount of the test sample was about 25 mL, which is quite a considerable amount for biosensor applications. Table 3 shows the analytical results after the possible size shrinking. It reveals that the resonant frequency increases, and the sensitivity increases much higher. By shrinking the size to use the test sample of 0.1 mL, the resonant frequency is 134.8 kHz and the corresponding absolute sensitivity is 299.2 Hz/(kg/m^3^). The frequency is relatively low compared with the previous method based on a mico-cantilever beam while the sensitivity is much higher (in [25], the resonant frequency was more than 160 kHz and the corresponding sensitivity was lower than 100 Hz/(kg/m^3^)). On the other hand, as disadvantages of the method presented in this paper, the appropriate miniaturized version (e.g., MEMS devices) of acoustic power source, sensors, and acoustic mediums will be needed to make miniaturized device work effectively.

In this paper, a principle and sensing scheme of a novel biosensor architecture based on Helmholtz resonance were suggested and verified. As future work, miniaturization and integration of the proposed HR architecture will be done with further optimization for commercial applications.

## 5. Conclusions

In this work, an indirect sensing methodology was proposed to measure an analyte concentration in a test solution by using Helmholtz resonance. The resonant frequency of the proposed Helmholtz resonator with a test solution inside was analytically derived. By setting up the experiment system and making glucose solutions with different concentrations, the experiment was carried out. The example application for measurement of glucose concentration in a test solution by using a Helmholtz resonator was analyzed and tested, revealing relatively higher absolute (2×) and relative (4×) sensitivities compared to the previous cantilever-based approaches. Measurements of practical glucose concentration levels of human blood were examined in an aqueous solution and the results showed good performance in a relatively low frequency range, demonstrating the possibility of measuring glucose concentration in human blood after miniaturization and integration of the proposed HR architecture.

## Figures and Tables

**Table 1:** Resonant frequencies obtained by analytical and experimental approaches.

**Glucose Concentration**	0 wt %	4.9 wt %	9.7 wt %	15.9 wt %	30.4 wt %
**Analytical Results (A)**	5597 Hz	5766 Hz	5829 Hz	5915 Hz	6132 Hz
**Experiment Results (B)**	5609 Hz	5797 Hz	5938 Hz	6123 Hz	6522 Hz
Relative Error (|A−B|/B*100%)	0.21%	0.53%	1.83%	3.40%	5.98%

**Table 2:** Parameters and resonant frequencies for different practical glucose levels.

Glucose Levels (mg/dL)	Density (kg/m^3^)	Sound Speed (m/s)	Resonant Frequency (Hz)	
			Analytical Results	Experiment Results
0	998	1479	5596	5609
72	998.25	1480.2	5601	5617
162	998.57	1481.6	5607	5623

**Table 3:** Resonant frequencies and sensitivities of the proposed Helmholtz resonator (HR) after miniaturization (analytical results).

**Amount of Test Sample**	25 mL	1 mL	0.1 mL
**Resonant Frequency**	5597 Hz	41.65 kHz	134.8 kHz
**Sensitivity**	11.99 Hz/(kg/m^3^)	92.67 Hz/(kg/m^3^)	299.2 Hz/(kg/m^3^)

## Supplementary Materials

The following are available online at https://www.mdpi.com/1424-8220/19/5/1127/s1, Figure S1: Spectrum of transmission loss of the air-filled HR, Table S1: Values of density and sound speed used in calculation, Figure S2: Tools for making the glucose solution, Table S2: Dimensions of the example model.
