# Supplementary material for: Measurement of an Analyte Concentration in Test Solution by Using Helmholtz Resonator for Biosensor Applications
Source: Sensors (Basel). 2019 Mar 5;19(5):1127. doi: 10.3390/s19051127 (PMC6427302; doi:10.3390/s19051127)
Supplement: Supplementary file 1 [file sensors-19-01127-s001.pdf]

Supplementary Material for
Measurement of an Analyte Concentration in Test Solution by Using Helmholtz Resonator for Biosensor Applications

Table 1. Values of density and sound speed used in calculation.

Glucose concentration	Density (kg/m^3)	Sound speed (m/s)
0 wt.% (Water)	998	1479
34.9 wt.%	1107	1654
4.9 wt.%	1012.1	1523.4
9.7 wt.%	1026.2	1540.3
15.9 wt.%	1044.9	1564.0
30.4 wt.%	1091.9	1630.1

Table S2: Dimensions of the example model.

Parameters	Duct		HR				Fluid	
	L_d	R_d	L_n	R_n	L_c	R_c	L_f	L_s
Values	100mm	15mm	10mm	5mm	20mm	20mm	2.4mm	7.6mm

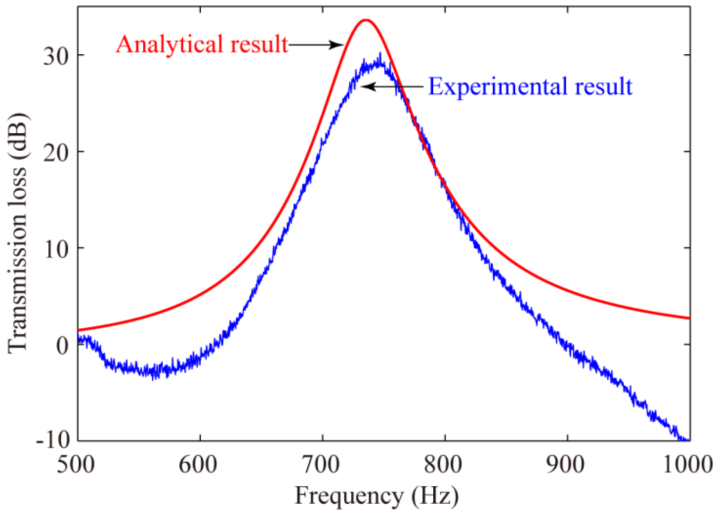

Figure S1: Spectrum of transmission loss of the air-filled HR

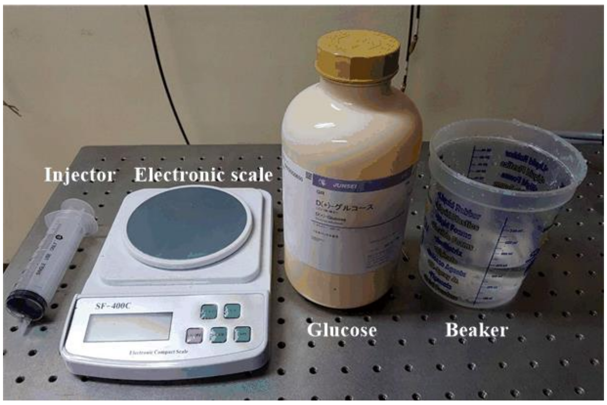

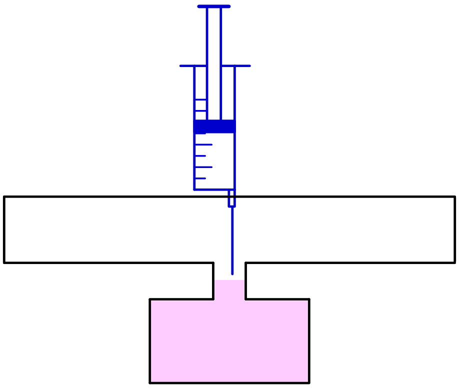

Figure S2: Tools for making the glucose solution

Derivation of the Resonant Frequency and Transmission Loss of the Proposed HR

A typical Helmholtz resonator (HR) structure is shown in Figure S3a, consisting of a chamber and a connected neck. Dynamic behavior of the HR can be effectively simplified using a mass–spring system analogy; a corresponding equivalent mass–spring model is shown in Figure S3a. Mass of fluid in the neck of the HR is equivalent to the mass, and adiabatically compressed volume of the fluid in chamber is equivalent to the spring. The incident pressure to the HR is equivalent to the input force to the simplified mass–spring system.

In this paper, for the purpose of measuring the substance concentration in the test solution, the HR was proposed as shown in Figure S3b.The test solution fills in the chamber and a portion of the neck of the HR, while the other (upper) part of neck is filled with another

fixed fluid.

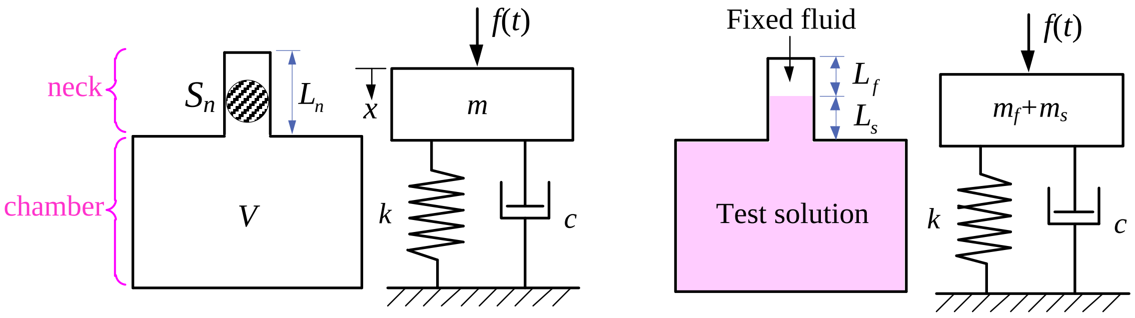

(a) A conventional HR model (b) Proposed HR for biosensor application

Figure S3: Structure of Helmholtz resonator (HR) and its equivalent mass-spring model.

For free vibration of the system, governing equation of motion is:

$$m\ddot{x} + kx = 0 \tag{s1}$$

The fluids trapped in the neck have total mass (m) of:

$$m = m_f + m_s = \rho_f S_n L'_f + \rho_s S_n L'_s \tag{s2}$$

where ρ_f and ρ_s are densities of fixed fluid and test solution, respectively, and S_n is the cross-sectional area of the neck. L'_f and L'_s are the equivalent lengths of the fixed fluid and test solution in the neck which are usually longer than L_f and L_s because of the radiation mass loadings. They can be computed as follows

$$L'_f = L_f + \alpha \tag{s3a}$$

$$L'_s = L_s + \beta \tag{s3b}$$

where α and β are the end correction lengths; they can be determined as $\alpha = 0.36\sqrt{S_n}$, $\beta = 0.48\sqrt{S_n}$.

To determine the stiffness (k) of the equivalent system, it is assumed that the fluid (including the fixed fluid and test solution in neck) is trapped in the neck by a sealed piston. If the piston is pushed by a distance ΔL, the volume of test solution in chamber will change as:

$$\Delta V = -S_n \Delta L \tag{s4}$$

Then the pressure generated by volume compression of the test solution in chamber is:

$$P = \kappa_s \frac{-\Delta V}{V} = \kappa_s \frac{S_n \Delta L}{V} \tag{s5}$$

where $\kappa_s = \rho_s c_s^2$ is the bulk modulus of the test solution, c_s is the sound speed in the test solution, and V is the volume of chamber.

Therefore, the stiffness (k) can be obtained as:

$$k = \frac{PS_n}{\Delta L} = \rho_s c_s^{\ 2} \frac{S_n^2}{V} \tag{s6}$$

Then the resonant frequency can be calculated by using:

$$f_{hr} = \frac{1}{2\pi}\sqrt{\frac{k}{m}} = \frac{c_s}{2\pi}\sqrt{\frac{\rho_s S_n}{\left(\rho_f L_f' + \rho_s L_s'\right)V}} \tag{s7}$$

The HR can function as a muffler when it is attached to a duct as a side branch, as depicted in Figure S4. The acoustic energy in the system is attenuated by fluid mass movement in the neck of HR, which is regulated by the equivalent stiffness of fluid in the chamber of HR. The most effective sound attenuation effect can be obtained at frequencies in the vicinity of the resonant frequency of HR described in eq (s7).

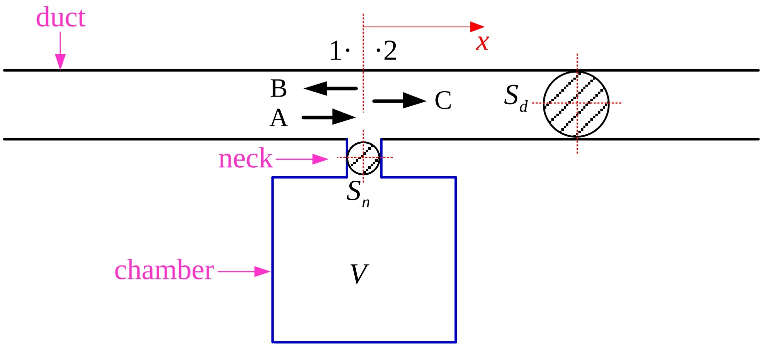

Figure S4: The duct with side-branch HR.

From the viewpoint of acoustic impedance, the HR reduces noise by impedance mismatch between the duct and the HR. Impedance mismatch causes reflection and attenuation of the incident acoustic energy in the HR's neck. In this situation, the basic assumption is that plane waves propagate along the duct and there are no reflected waves from downstream of the duct because of the absence of mean flow. The acoustic impedance of HR can be written as:

$$Z_H = R_H + j\left(\omega L_H - \frac{1}{\omega C_H}\right) \tag{s8}$$

where R_H is the specific acoustic resistance of the HR, $\omega L_H - \frac{1}{\omega C_H}$ is the specific acoustic reactance of the HR, and ω is the frequency of acoustic wave.

If the acoustic radiation of the HR is assumed to be thus in the case of an open-ended duct, then we get:

$$R_H = \rho_f c_f K^2 \frac{S_n^2}{2\pi} \tag{s9}$$

where K is the wave number calculated by ω / c_f, and c_f is the sound speed in fixed fluid.

The acoustic inertance L_H and acoustic compliance C_H are:

$$L_H = \frac{\rho_f L'_f + \rho_s L'_s}{S_n} \qquad C_H = \frac{V}{\kappa_s} \tag{s10}$$

The sound pressure (P_1, P_2) and volume velocity (U_1, U_2) of point 1 and point 2 can be expressed as:

$$P_1 = Ae^{-jKx} + Be^{jKx} \qquad P_2 = Ce^{-jKx} \tag{s11}$$

$$U_1 = \frac{1}{Z_D}\left(Ae^{-jKx} - Be^{jKx}\right) \qquad U_2 = \frac{1}{Z_D} Ce^{-jKx} \tag{s12}$$

where A, B, and C are the magnitudes of the incident wave, reflected wave, and transmitted wave, respectively, Z_D is the acoustic impedance of the duct, and $Z_D = \rho_f c_f / S_d$, where S_d is the cross-sectional area of duct.

Considering the continuity of the sound pressure and volume velocity at the duct-neck interface, the relationship of pressure and volume velocity between point 1 and point 2 can be expressed by the transfer matrix as:

$$\begin{pmatrix} P_1 \\ U_1 \end{pmatrix} = \begin{bmatrix} 1 & 0 \\ \frac{1}{Z_H} & 1 \end{bmatrix} \begin{pmatrix} P_2 \\ U_2 \end{pmatrix} = \begin{bmatrix} T_{11} & T_{12} \\ T_{21} & T_{22} \end{bmatrix} \begin{pmatrix} P_2 \\ U_2 \end{pmatrix} \tag{s13}$$

Then transmission loss of the side-branch HR mounted on a duct can be obtained by combining eq (s11), eq (s12), and eq (s13) as:

$$TL = 20\log_{10}\left(\frac{A}{C}\right) = 20\log_{10}\left|\frac{1}{2}\left(T_{11} + \frac{1}{Z_D}T_{12} + Z_D T_{21} + T_{22}\right)\right| = 20\log_{10}\left(\left|1 + \frac{\rho_f c_f}{2S_d}\frac{1}{Z_H}\right|\right) \tag{s14}$$
